# Characterization and regulation mechanism analysis of ubiquitin-conjugating family genes in strawberry reveals a potential role in fruit ripening

**DOI:** 10.1186/s12870-021-03421-8

**Published:** 2022-01-19

**Authors:** Mengyao Li, Liangxin Wang, Yiting Liu, Yuanxiu Lin, Yunting Zhang, Yu Long, Chuanying Luo, Yong Zhang, Qing Chen, Pinwen Chen, Yan Wang, Xiaorong Wang, Haoru Tang, Ya Luo

**Affiliations:** 1 College of Horticulture, Sichuan Agricultural University, Chengdu, 611130 China; 2 Institute of Pomology and Olericulture, Sichuan Agricultural University, Chengdu, 611130 China; 3 Departmental and Municipal Co-construction of Crops Genetic Improvement of Hill Land Key Laboratory of Sichuan, Nanchong, 637000 China

**Keywords:** Strawberry, Ubiquitin-conjugating enzymes, Phylogenetic analysis, Expression patterns analysis, Fruit ripening

## Abstract

**Background:**

E2 ubiquitin-conjugating (UBC) enzymes are an integral component of the ubiquitin proteasome system that play an important role in plant development, growth, and external stress responses. Several UBC genes have been identified in various plants. However, no studies exploring the functions of UBC genes in regulating fruit of strawberry have been reported. In the present study, a systematic analysis of the entire UBC family members were conducted in the genome of strawberry (*Fragaria* ×*ananassa*) based on bioinformatics method, and the gene functioning in strawberry ripening was explored.

**Results:**

A total of 191 UBC genes were identified in the genome of cultivated strawberry. These genes were unevenly distributed across the 28 chromosomes from the 4 subgenomes of cultivated strawberry, ranging from 3 to 11 genes per chromosome. Moreover, the expansion of FaUBC genes in strawberry was mainly driven by WGD. All the FaUBC genes were clarified into 13 groups and most of them were included in the group VI. The gene structure analysis showed that the number of exons varied from 1 to 23, and the structure of genes had few differences within the same groups but a distinction in different groups. Identification of the cis-acting elements of the promoter revealed multiple regulatory elements that responded to plant growth and development, phytohormone responsive, and abiotic and biotic stress. Data from functional annotation indicated that FaUBC genes play a role in a variety of biological processes. The RNA-seq data showed that FaUBC genes displayed different expression pattern during the fruit ripening process and clarified into 6 clusters. In particular, cluster 3 exhibiting a sudden expression increase in the turning red stage were speculated to be involved in fruit ripening. Hence, two FaUBC genes (*FaUBC76* and *FaUBC78*) were selected for gene function analysis by transient over-expression method. The results indicated that *FaUBC76* has a positive effect on the fruit development and ripening in strawberry by up-regulating accumulation of anthocyanins. Moreover, expression of some maturity-related genes were also significantly increased, further supporting a role for *FaUBC76* in the regulation of fruit ripening or softening. On the contrary, the overexpression of *FaUBC78* significantly increased the firmness of strawberry fruit, indicating that *FaUBC78* had a positive role in inhibiting the decrease of strawberry fruit firmness.

**Conclusion:**

Our study not only provide comprehensive information on system evolution and function on UBC genes, but also give a new insight into explore the roles of FaUBC genes in the regulation of strawberry ripening.

**Supplementary Information:**

The online version contains supplementary material available at 10.1186/s12870-021-03421-8.

## Introduction

As a ubiquitous post-translational modification for eukaryotes, ubiquitination is involved in many cellular processes, including hormone signaling transduction [1], apoptosis [2], and biotic and abiotic stresses stresses [3]. Protein ubiquitination requires the concerted action of ubiquitin-activating enzyme (E1), ubiquitin-conjugating enzyme (E2) and ubiquitin ligase (E3). In the initial step, the E1 transfers ubiquitin (Ub) to the active site cysteine of E2 in an ATP-dependent manner and forms an E2-Ub intermediate. Subsequently, the E2-Ub conjugate interacts with an E3 to ligate Ub to a lysine side chain of the target protein and build mono- or poly-ubiquitin chains, then the target protein is degraded or modified to perform different functions [4].

The E2 genes exist as a multi-gene family in higher plants, which have been identified in rice [5], tomato [6], banana [7] and potato [8]. Being a key enzyme of ubiquitination process, E2 protein has a conserved UBC domain containing 150-200 amino acids in length [9]. Ubiquitin-conjugating enzyme plays a crucial role in plant growth and development [6], and also participates in environmental stresses [10, 11], immune response [12, 13], DNA damage and repair [14]. In Arabidopsis, *AtUBC32* is an endoplasmic reticulum-associated degradation (ERAD) component that functions in brassinosteroid-mediated salt stress tolerance [15]; *AtUBC2* is implicated in repression of flowering [16]; *AtUBC13* was involved in epidermal cell differentiation and iron deficiency responses [17, 18]. A tomato UBC13-type homologous protein, FNI3, is involved in the regulation of the immune response [19]. The expression levels of *Cucumis melo* UBC enzyme (*CmUBC*) were increased under drought and salt stress in melon [20]. Wheat defense systems against *Zymoseptoria tritici* can be regulated by *Triticum aestivum* ubiquitin-conjugating enzymes 4 (*TaU4*) [21]. The *Arabidopsis UBC22*-knockout mutants reduced the length and seed number of siliques, and caused an early arrest of nearly 90% ovules development [22]. It was reported that the regulator RIN (Ripening-inhibitor) of tomato fruit ripening could directly combine with the E2 promoter region, and the fruit color was changed after silencing the E2 gene [6].

Strawberry is a nutritionally important fruit as well as an ideal model plant for studying non-climacteric fruits. There is a growing recognition that fruit ripening regulation can meet the supply-demand balances of market to some extent. To regulate strawberry fruit development and ripening, previous researchers have mainly focused on abscisic acid (ABA) [23], auxin [24, 25] and sucrose [26]. Genome-wide analysis of the UBC genes in cultivated strawberry would be necessary for strawberry fruit development and ripening research. In this study, we performed a genome-wide analysis of UBC genes in strawberry with a focus on gene structure, evolutionary analysis, and expression abundance. Moreover, the role of E2 genes on strawberry fruit development and ripening were analyzed.

Studies have shown that ABA is a key hormone in the regulation of fruit ripening of strawberry and other non-climacteric fruit. ABA can promote the expression of genes related to strawberry fruit ripening [27]. At the same time, ABA can also promote the expression of other genes related to coloring and softening of non-respiratory climacteric fruits, such as chalcone synthase (*CHI*) and expansin (*EXP*) genes [28]. Moreover, the reception and transduction of ABA signals are regulated by protein ubiquitin modification [29, 30]. It reported that the *ubc32* mutant of E2 protein gene showed an insensitive phenotype to ABA during and after seed germination, and *UBC32* was a positive regulator of ABA signal [31]. Current studies have confirmed that ABA plays an important role in strawberry fruit ripening. ABA is closely related to ubiquitination, whereas ubiquitin modification plays a role in strawberry fruit ripening has not been reported. In view of this, this study intends to explore the function of E2 in strawberry fruit ripening, in order to enrich the regulatory network of strawberry fruit ripening.

## Results

### Genome-wide identification, and characteristics of FaUBC genes in strawberry

**Fig. 1:**
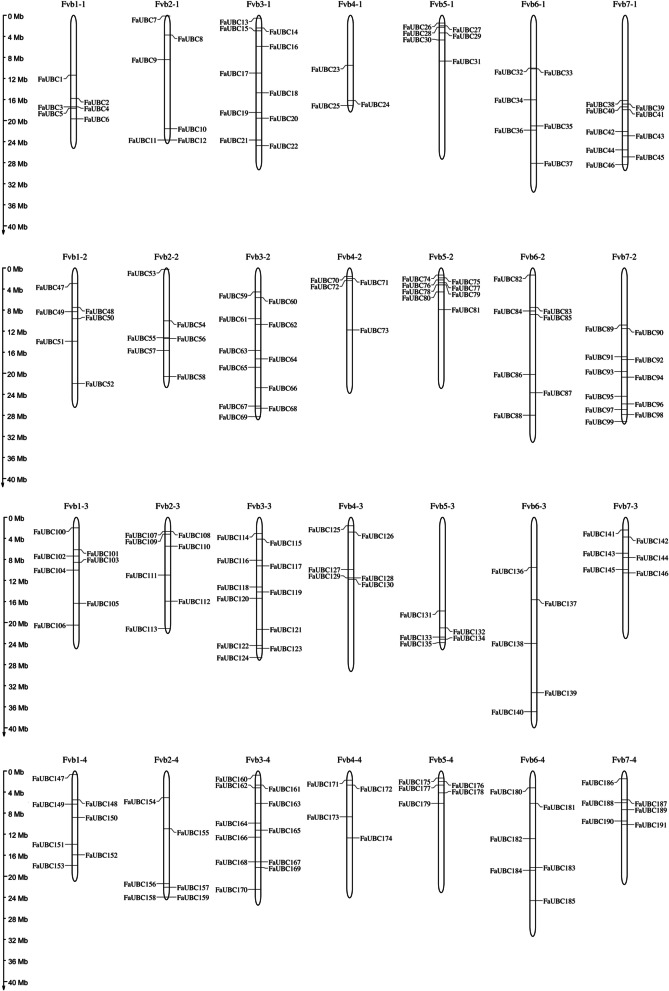
Chromosomal distribution of UBC genes in the strawberry genome. Chromosome numbers are provided at the top of each chromosome. Scale is in megabases (Mb)

Based on the genome searching and domain confirmation, a total of 191 FaUBC genes were identified in the genome of cultivated strawberry. All the identified FaUBC genes were renamed according to the order of their chromosome location (Fig. 1). As the result showed, 191 FaUBC genes were unevenly distributed across the 28 chromosomes in the 4 subgenomes of cultivated strawberry, ranging from 3 to 11 genes per chromosome. A maximum 11 FaUBC genes was located on chromosome 7 and 3 from the second subgenome (Fvb7-2 and Fvb3-2), as well as the chromosome 3 from the third and fourth subgenomes (Fvb3-3 and Fvb3-4), followed by 10 genes on chromosome 3 from the first subgenome (Fvb3-1). On the contrary, the minimum number of FaUBC genes (3 members) was distributed on chromosome 4 from the first subgenome (Fvb4-1).

The physicochemical properties of the 191 FaUBC proteins were also analyzed and shown in Table S1. The number of amino acids varied from 67 to 1921, and most of them (145 out of 191, 75.9%) were from 100 to 300. The protein molecular weights (MW) were from 7.478 to 216.883 KDa, and the isoelectric points (pI) were concentrated from 3.88 to 10.55. Except for FaUBC134 and FaUBC106, all of the other FaUBC genes didn’t contain a signal peptide, which is essential for secreted proteins. Most of the FaUBC genes were predicted to be located in the cytoplasmic, chloroplast and nuclear, also some FaUB*C* genes were involved in mitochondrial, endoplasmic reticulum, plasma, vacuole, peroxisome and extracellular. Notably, there are some FaUBC genes were predicted to be dual-located, for instance, *FaUBC35* and *FaUBC139* were located in cytoplasmic and peroxisome, *FaUBC125* was located in both nuclear and cytoplasmic, while *FaUBC134* was located in extracellular, vacuole or endoplasmic reticulum.

### Classification and structural analysis of FaUBC proteins in strawberry

**Fig. 2:**
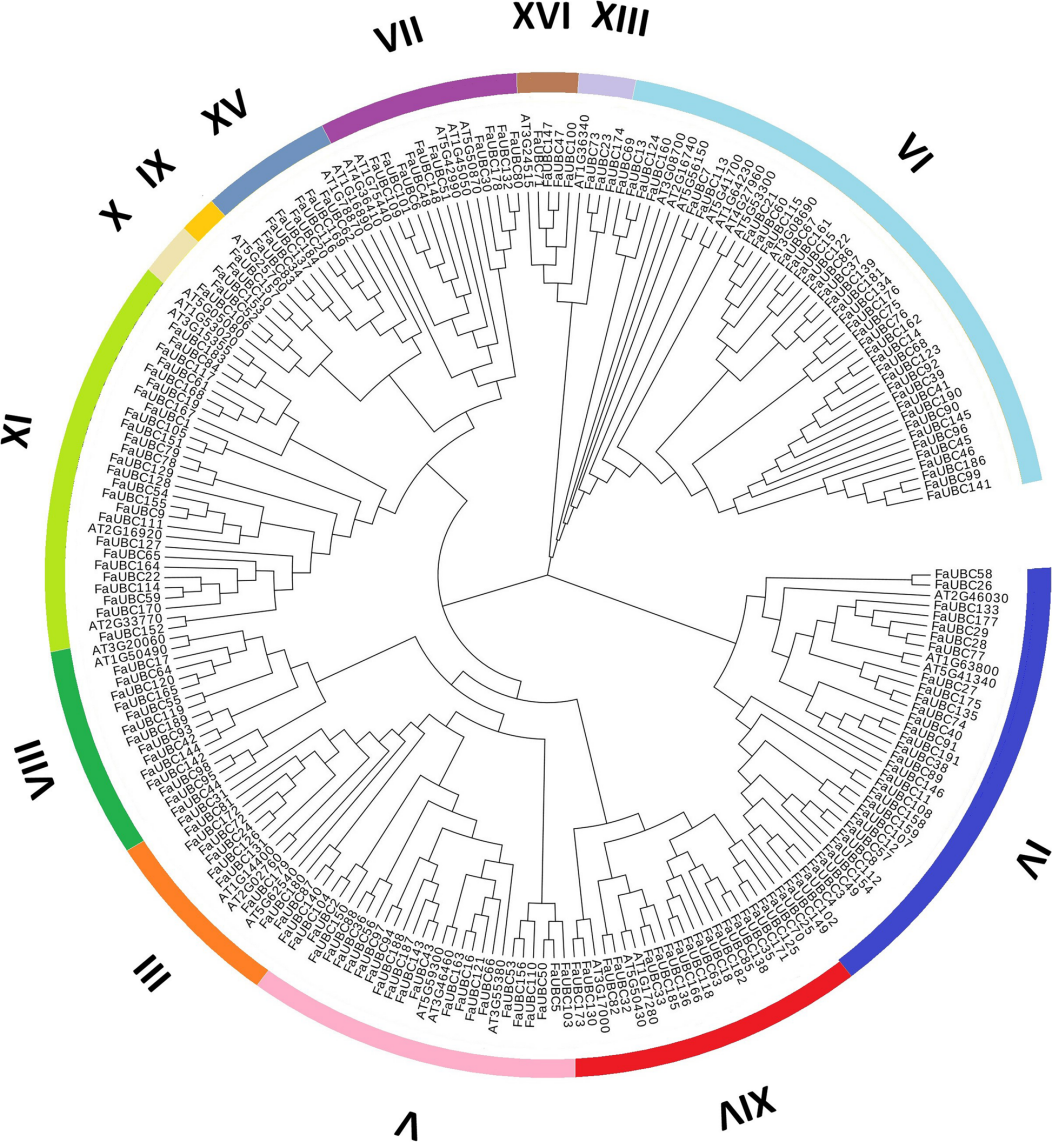
Phylogenetic analysis and classification of FaUBC proteins among strawberry and Arabidopsis by MEGA7.0. The phylogenetic tree was constructed using the neighbor-joining method with 1000 bootstrap replicates. The tree was divided into 13 groups and each group is shown in different colors

According to the classification of UBCs in Arabidopsis, all the FaUBC proteins were classified into 13 groups (Fig. 2), most (37 members) of which were included in the group VI, followed by group IV (32 members). The gene structure was also investigated (Fig. S1): the number of exons varied from 1 to 23, and the structure of genes had few differences within the same groups but a distinction in different groups. In addition, the motif analysis showed that 10 motifs were found in the amino acids sequences of FaUBC proteins (Fig. S1). Motif 1 is Ubiquitin-conjugating domain and found in all of the FaUBC sequences. Motif 10 is specific in group XIV, while motif 9 encoding an Ubiquitin-conjugating domain and motif 8 is specific in group XI. The motifs 5 and 2 were also detected as Ubiquitin-conjugating domain and found in most of the FaUBC sequences.

### Synteny and evolutionary analysis of FaUBC genes in strawberry

**Fig. 3:**
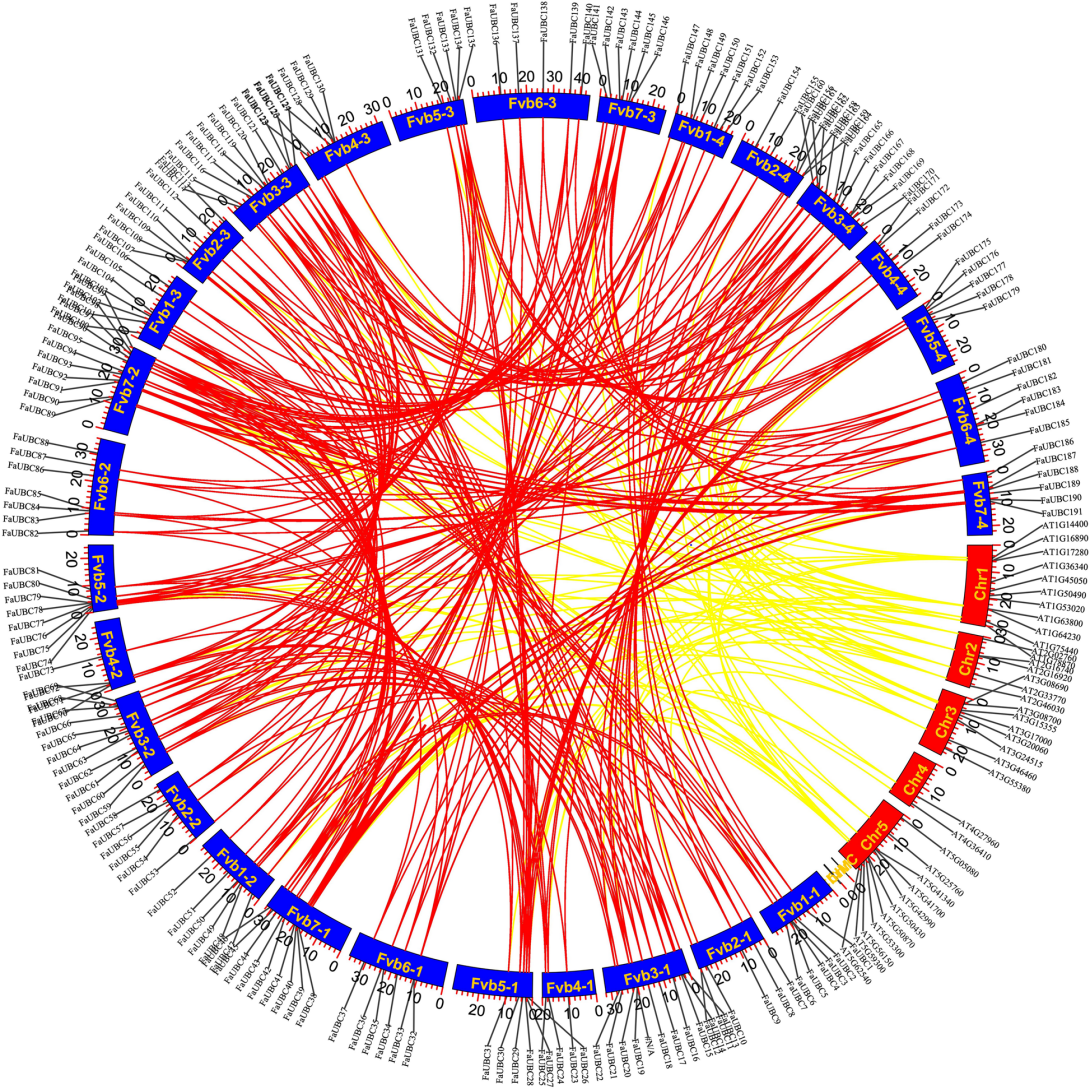
Synteny analysis of FaUBC genes and AtUBC genes. The chromosomes of strawberry and Arabidopsis were showed as blue and red colors as circular, respectively. The position of UBC genes were marked as black line on the chromosome. Gene pairs in the strawberry/strawberry and strawberry/Arabidopsis with synteny relationships were represented with red and yellow curves, respectively

The synteny relationship between Arabidopsis AtUBC genes and strawberry FaUBC genes was investigated to explore the origin and evolutionary process (Fig. 3). A total of 233 pairs of UBC genes were identified as collinear pairs in strawberry, while 78 collinear pairs were identified between Arabidopsis and strawberry (Table S2). Subsequently, the origins of duplication events of FaUBC genes in strawberry were detected using MCScanX package. The result showed that five types of duplication events were detected, including whole genome duplication (WGD) or segmental (WGD/segmental), Dispersed, Tandem, Singleton and Proximal duplication (Table S1). Most of which were WGD/segmental, followed by Dispersed with a number of 16 genes. Only *FaUBC3* and *FaUBC4*, *FaUBC128* and *FaUBC129* were duplicated from Tandem duplication. In addition, *FaUBC58* was detected in Singleton duplication event, while *FaUBC78*, *FaUBC79* and *FaUBC168* were duplicated from Proximal. These results suggested that the expansion of FaUBC genes in strawberry was mainly driven by WGD. Additionally, the number of non-synonymous substitutions per non-synonymous sites (Ka), the number of synonymous substitutions per synonymous sites (Ks) and the Ka/Ks ratio for paralogous gene pairs of FaUBC genes were calculated (Table S3). The results showed that the Ka and Ks ranged from 0 to 4.1 and 0.006-3.617 for identified paralogs respectively, the ratio of Ka/Ks ranged from 0 to 2.09. Most of the protein-coding genes have a Ka/Ks ratio of less than one, and only 23 gene pairs exhibited positive selection with a Ka/Ks ratio greater than one.

**Fig. 4:**
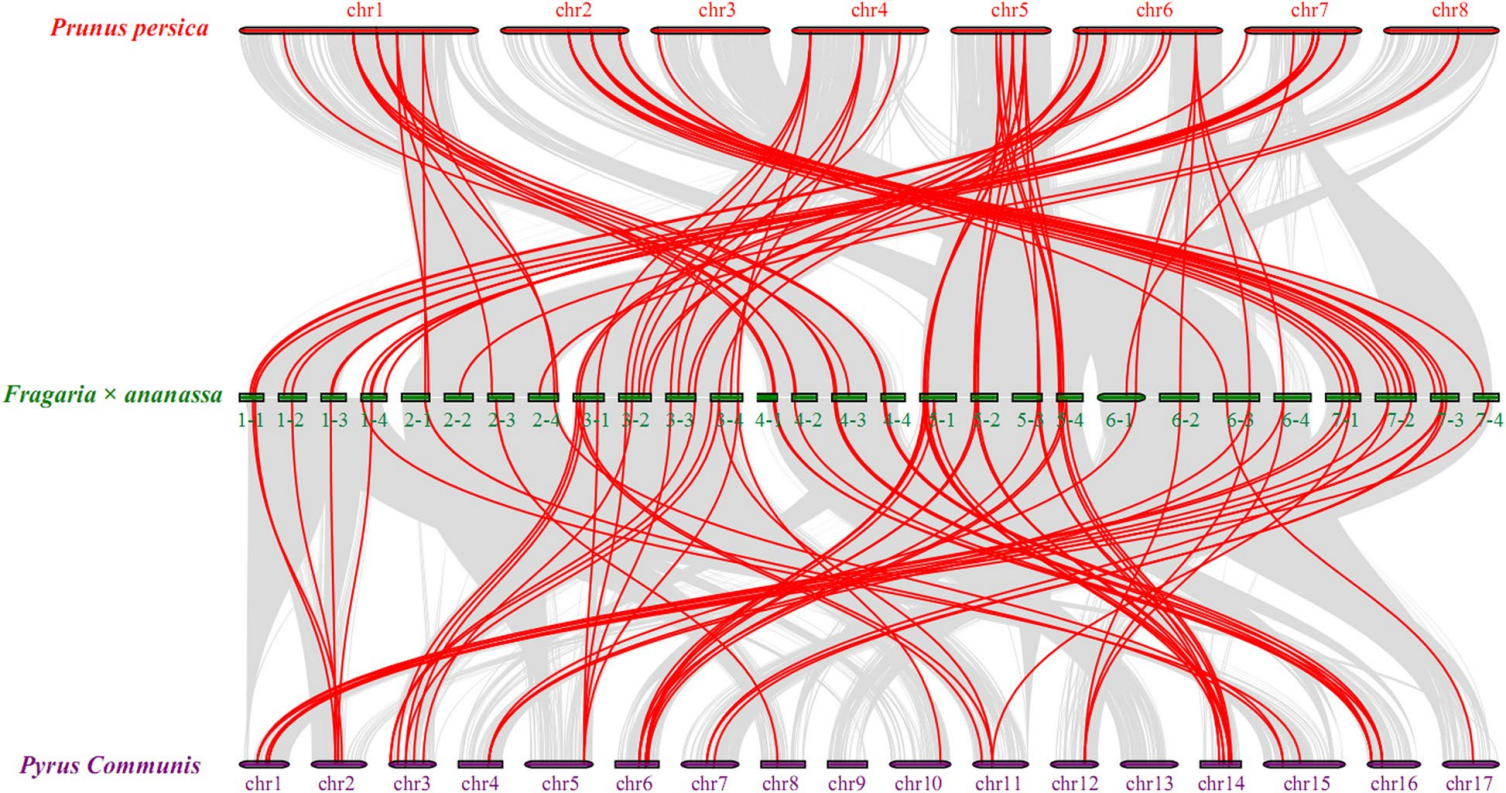
Analysis of collinearity among three Rosaceae species. The red, green, and purple circular rectangles denote the chromosomes of peach (*Prunus persica*), strawberry (*Fragaria×ananassa*), and pear (*Pyrus communis*), respectively. The gray curves connect protein-coding genes in the synteny blocks, and the red curves represent gene pairs that are collinear with UBC genes

To illustrate the evolutionary relationship of UBC genes, a collinearity analysis of UBC genes among peach (*Prunus persica*), strawberry (*Fragaria×ananassa*), and pear (*Pyrus communis*) was constructed. The result revealed 69 and 74 UBC paralogous gene pairs in strawberry/peach and strawberry/pear, respectively (Fig. 4), and the ratio of Ka/Ks for each gene pair was analyzed (Table S4). According to the value of Ka and Ks, all the Ka/Ks ratios were lower than one, indicating that these paralogous gene pairs were under a strong purifying selection during evolution.

### Analysis of the cis-acting elements of the FaUBC genes

**Fig. 5:**
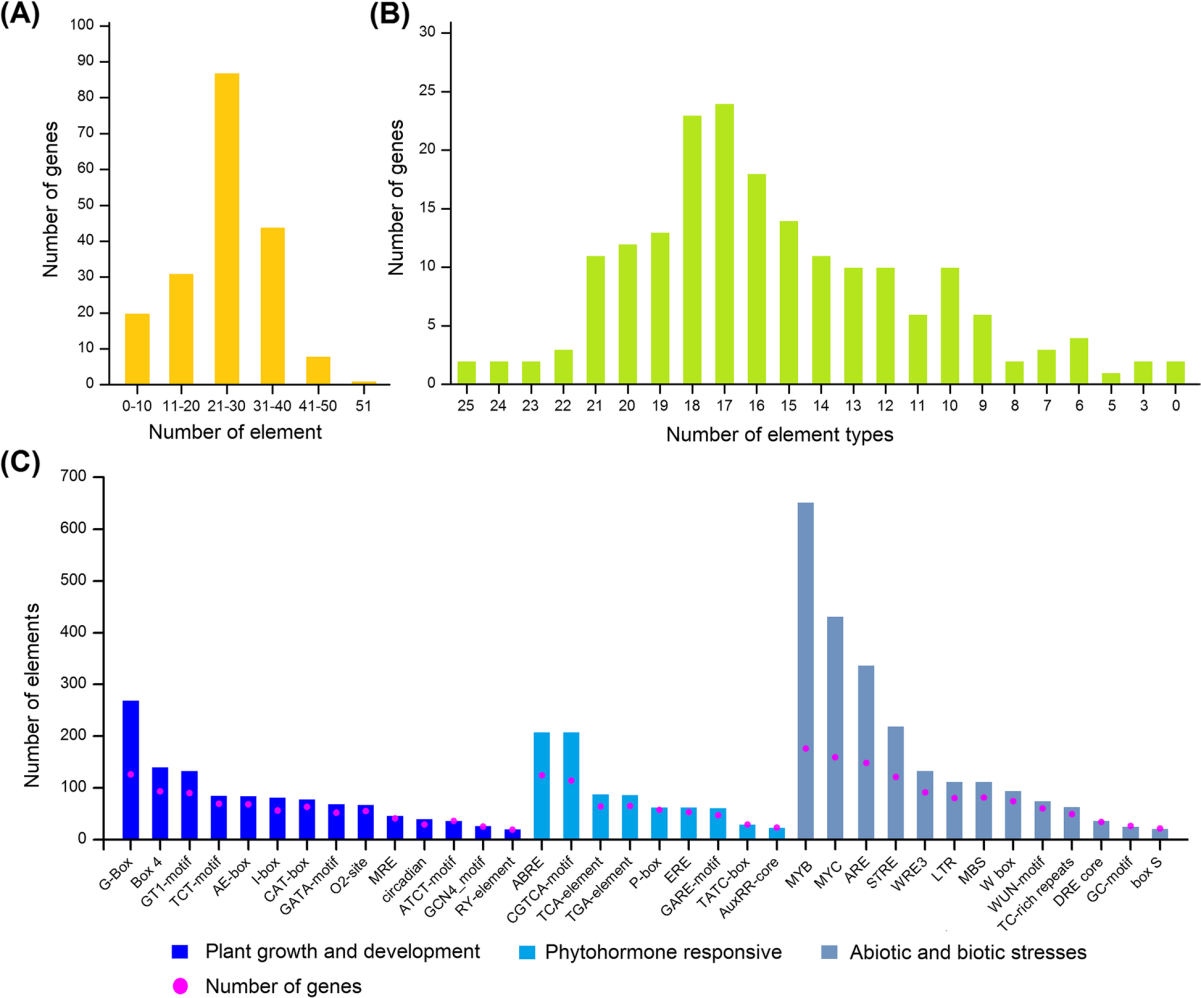
Analysis of cis regulatory elements from promoter region of the FaUBC genes. **A** Distribution of element number in FaUBC genes. **B** Distribution of number of element types in FaUBC genes. **C** Cis regulatory elements are divided into three classes: based on functional annotation: plant growth and development, phytohormone responsive, and abiotic and biotic stress

To further predict the function of the FaUBC genes, the cis regulatory elements in the UBC promoter regions were analyzed. The results showed that in addition to the basic core sequences TATA-box and CAAT-box which are unique to higher plant promoters, a total of 4642 elements belong to 76 cis element types were identified in 191 UBC genes (Table S5). Most genes contain 21-30 cis elements, among them, *FaUBC176* contains the largest number of elements, with 53 elements, followed by *FaUBC88*, *FaUBC29*, *FaUBC76*, and *FaUBC28* (Fig. 5A). The element types contained in each UBC promoter also vary greatly, most of which are in 9-21 types (Fig. 5B). Based on the functional annotation, all the elements were grouped into three major classes: plant growth and development, phytohormone responsive, and abiotic and biotic stress (Fig. 5C). A total of 126, 93 and 90 FaUBC members were observed to contain G-box, Box 4, and GT1-motif that are involved in light response during plant growth and development, respectively, indicating that they may be important in light response and photosynthetic product accumulation. Among the hormone-related elements, 239 ABA response elements (ABRE), 211 methyl jasmonate response elements (CGTCA-motif), and salicylic acid response elements (TCA-element) were found in multiple UBC genes. In addition, drought response elements (MYB and MYC), anaerobic induction (ARE), and stress response elements (STRE) were also detected in the promoters of multiple UBC genes.

### Functional annotation of FaUBC genes

**Fig. 6:**
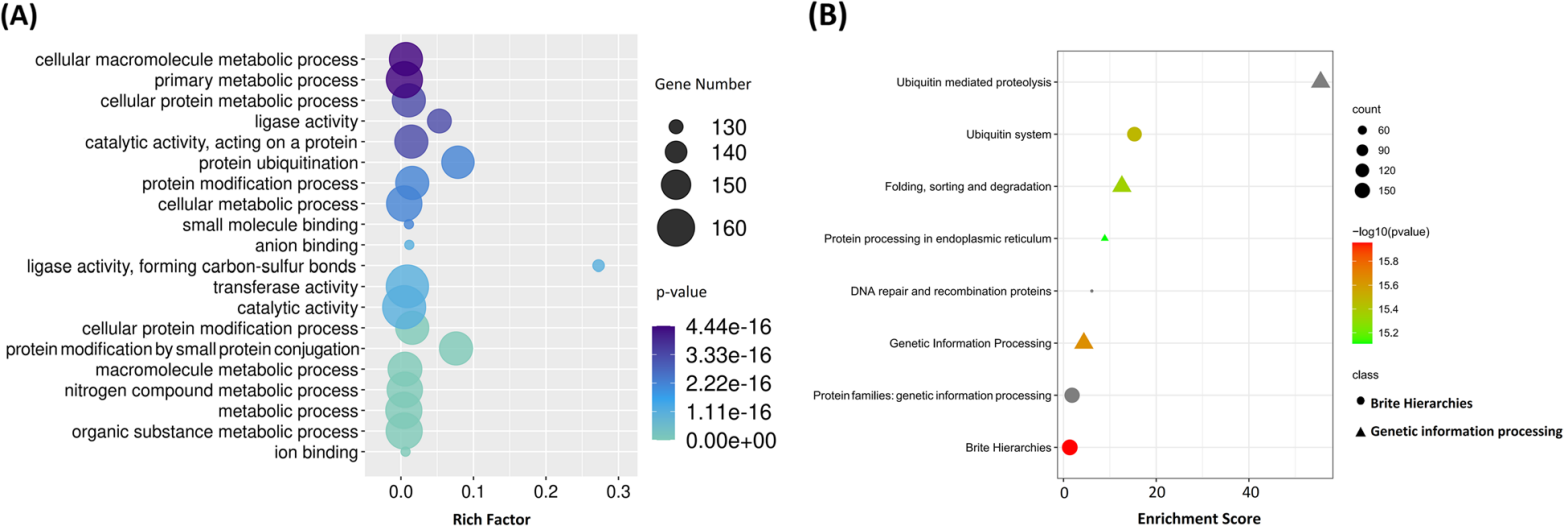
Functional annotation of FaUBC genes. **A** The top 20 of GO enrichment function annotation. **B** KEGG pathway enrichment analysis

The GO functional analysis of all FaUBC genes yielded 161 GO function terms (*p*-value < 0.05), including 122 biological process (BP) entries, 10 cell component (CC) entries, and 29 molecular function (MF) entries (Table S6). The GO terms “catalytic activity” and “transferase activity” were described the greatest number of genes with 168 and 167 members, and “protein ubiquitination”, “protein modification by small protein conjugation”, “ubiquitin-like protein transferase activity”, “ubiquitin activating enzyme activity”, “ubiquitin-protein transferase activity” and “ubiquitin conjugating enzyme activity” were also significantly enriched (Fig. 6A and Table S6). KEGG enrichment analysis results showed that 177 FaUBC genes were significantly enriched to 8 KEGG pathways (*p*-value < 0.05) (Fig. 6B and Table S7). Among them, 155 and 149 FaUBC genes were enriched into “Ubiquitin system” and “Ubiquitin mediated proteolysis” pathways, respectively. These annotations suggested that *FaUBC* genes are involved in a variety of biological processes.

### Protein-protein interaction network prediction of FaUBC proteins

**Fig. 7:**
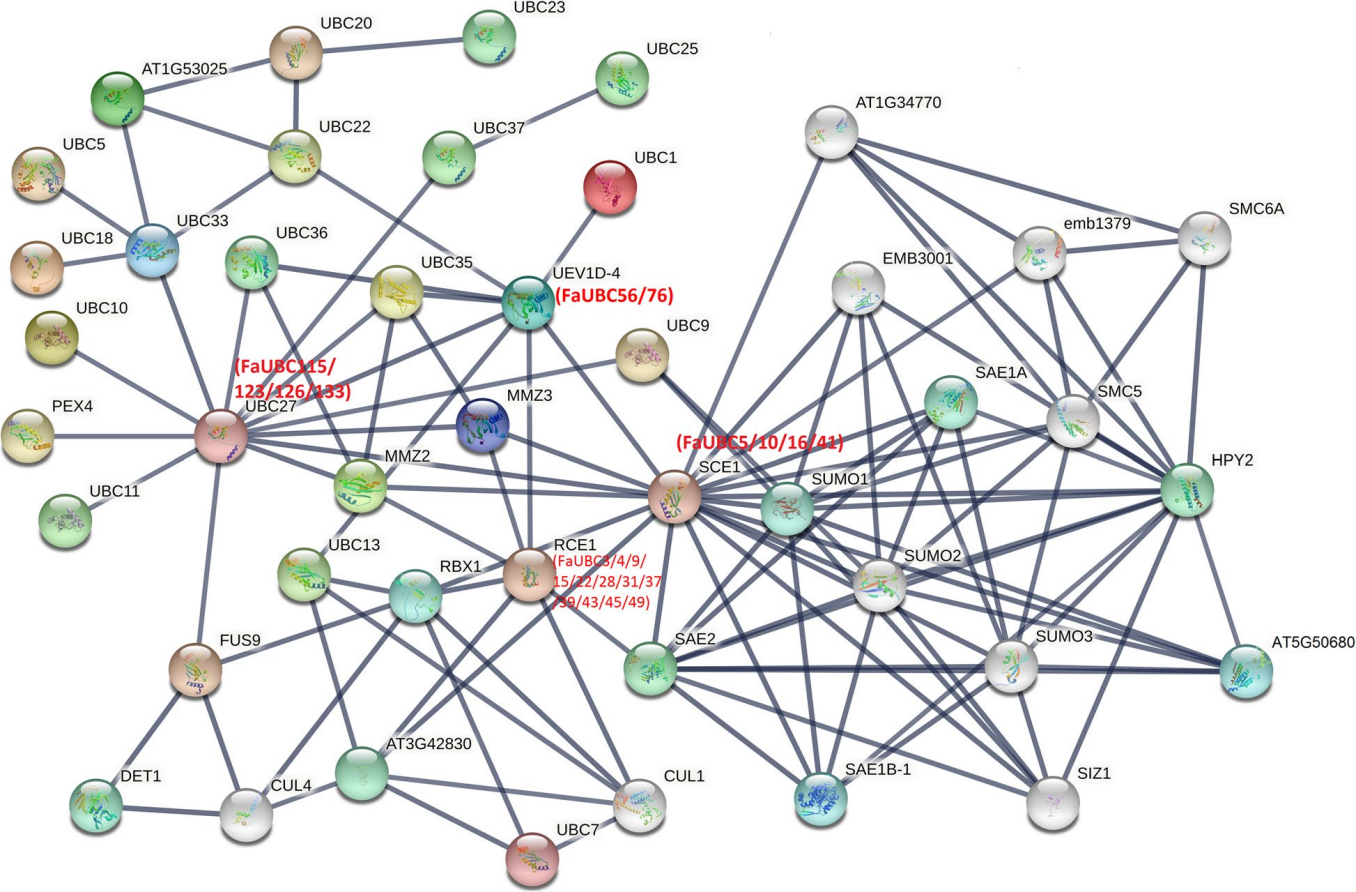
Interaction network of FaUBC genes based on the orthologs in Arabidopsis. The interaction network is based on the Arabidopsis model. Details Annotation of associated Arabidopsis and strawberry UBC proteins are presented in Supplementary Table 7

To predict the molecular interactions of FaUBC proteins, a protein-protein interactional analysis was performed based on their orthologues AtUBC proteins in Arabidopsis. Of the 191 FaUBC proteins, 176 (92.1%) displayed homology to 28 AtUBC proteins from Arabidopsis (Table S8). As illustrated in Fig. 7, SCE1 (homolog of FaUBC5/10/16/41) showed interactions with 19 proteins, including several UBC proteins (such as UBC27 and UEVID-4) and other proteins in strawberry like RING-H2 protein (RBX1), SUMO-activating enzymes (SAE1B-1 and SAE2), and small ubiquitin-related modifier proteins (SUMO1, SUMO2 and SUMO3). It is also predicted that UBC27 (homolog of FaUBC115/123/126/133) can interact with 13 proteins. UEVID-4 (homolog of FaUBC56/76) also may interact directly with UBC proteins like UBC1, UBC13, UBC27, UBC35, and UBC36. In addition, RCE1 (homolog of FaUBC3/4/9/15/22/28/31/37/39/43/45/49) showed significant correlations with RBX1, SAE2 and CUL1 (Cullin protein).

### Transcript abundance analysis of FaUBC genes in strawberry

**Fig. 8:**
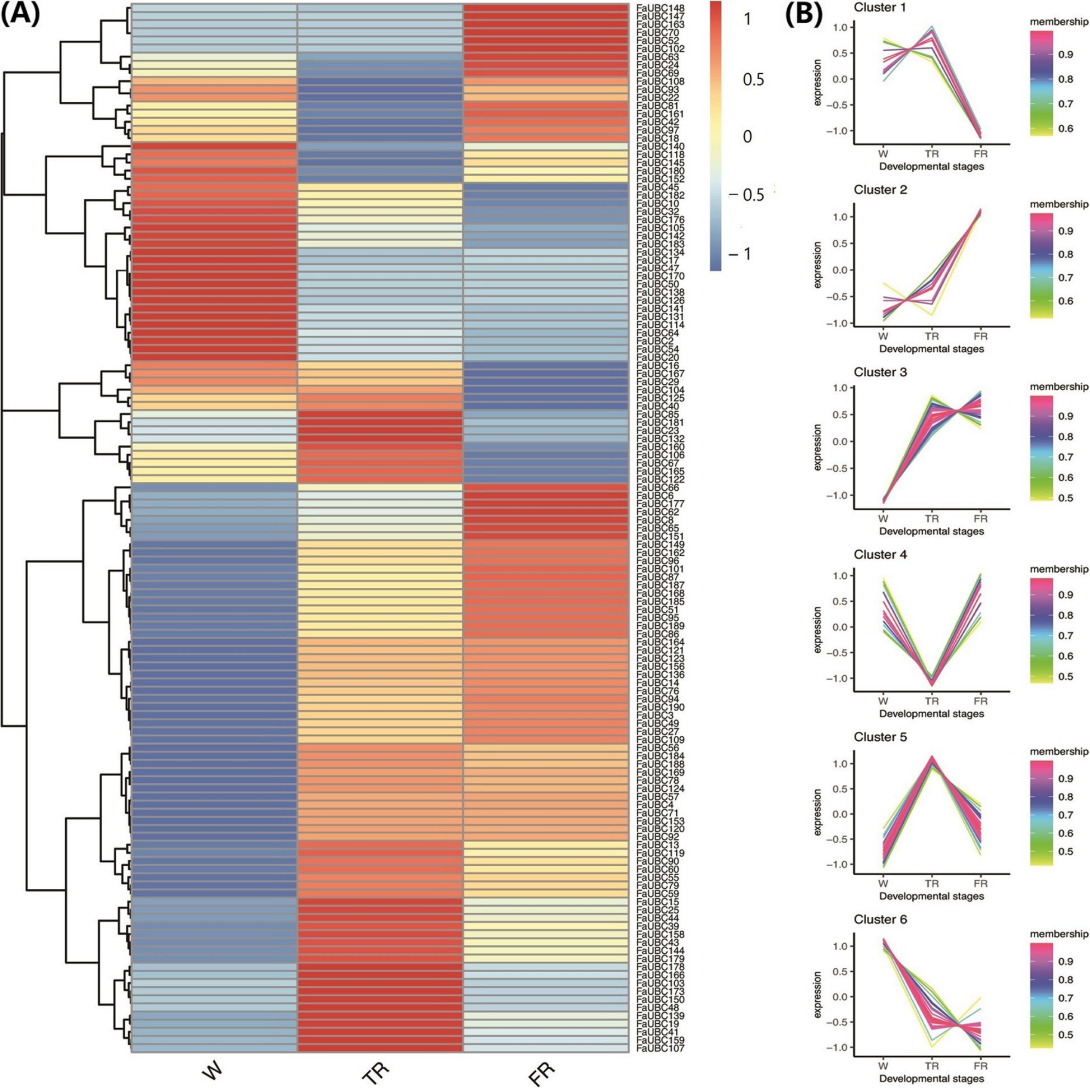
Transcript abundance of FaUBC genes in different treatments. **A** Heatmap of gene transcript abundance based on transcriptome data. W: white; TR: turning red; FR: full red. **B** Clustering of gene expression trends

To investigate the roles of FaUBC genes in the ripening of strawberry, the RNAseq-based expression was assessed. As illustrated in Fig. 8A, different FaUBC genes comprise different expression pattern during the fruit ripening process. Most genes were highly expressed in TR and FR stages. In general, according to their expression patterns, all the FaUBC genes could be clarified into 6 clusters as shown in Fig. 8B. Among them, 14 genes belonged in cluster 2 showing a gradual increasing of expression during fruit ripening, and 41 members in cluster 3 exhibiting a sudden expression increase in the TR stage were speculated to be involved in fruit ripening. All the other genes involved in the other clusters were listed in Table S9.

### Validation of the function of *FaUBC76* and *FaUBC78* in strawberry fruit ripening

**Fig. 9:**
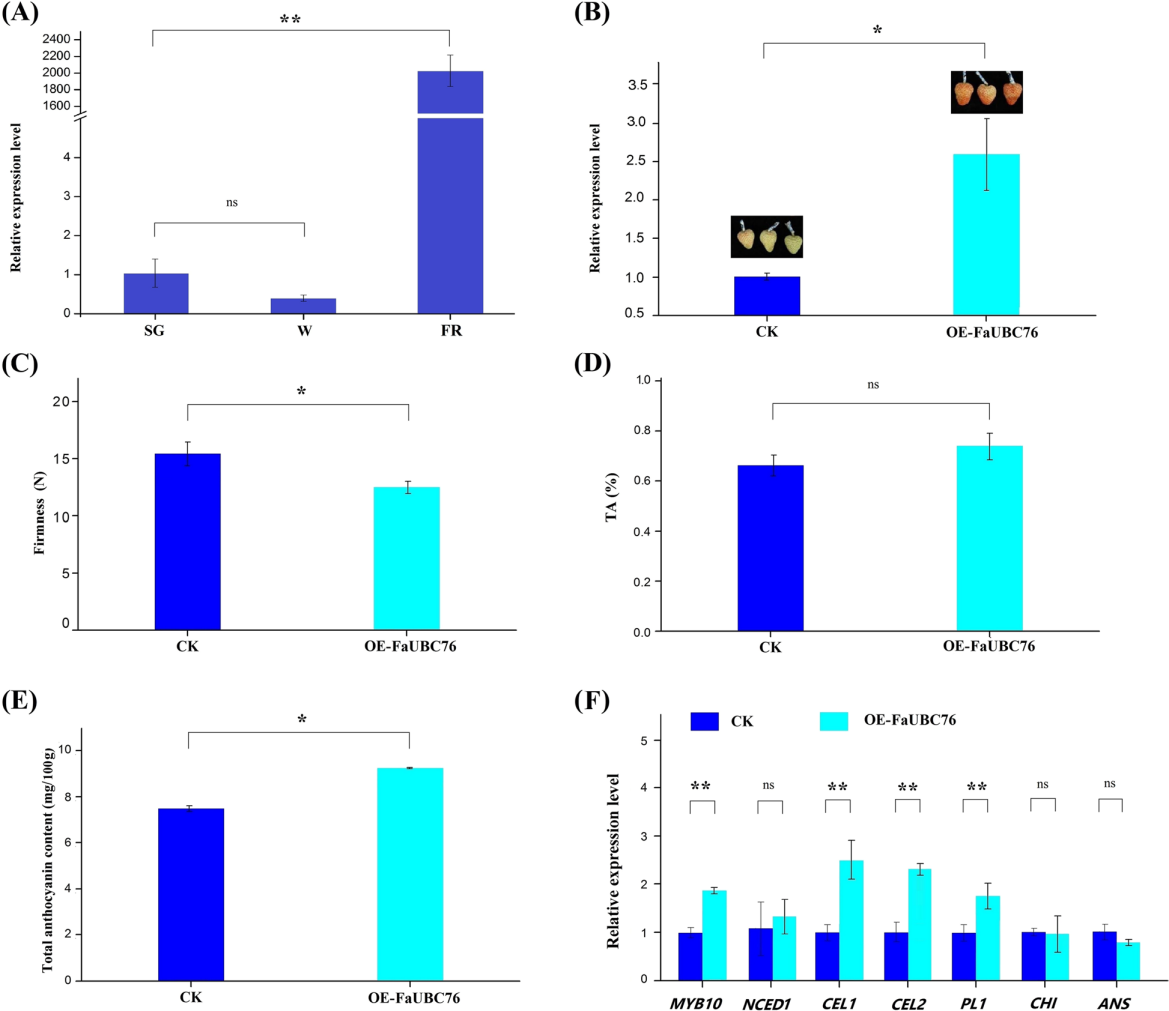
Overexpression for the *FaUBC76* gene in strawberry fruits. The injected fruits were harvested 5 days after injection. **A** RT-qPCR analysis of the relative expression level of *FaUBC76* at small green (SG), white (W) and partial red (PR) stages. **B** Transcript levels of *FaUBC76* in the control and overexpression fruit by RT-qPCR. **C**-**E** Fruit firmness, titratable acid content, anthocyanin content in the control and *FaUBC76* overexpression fruit. **F** Transcript levels of ripening-related genes in the control and *FaUBC76* overexpression fruits. Data are expressed as the mean ± SD of three replicates, and each repeat included 10 fruit individuals. Asterisks indicate significant differences (*P < 0.05, **P < 0.01, ns *P* > 0.05) between the means of the overexpression fruits and the control group

**Fig. 10:**
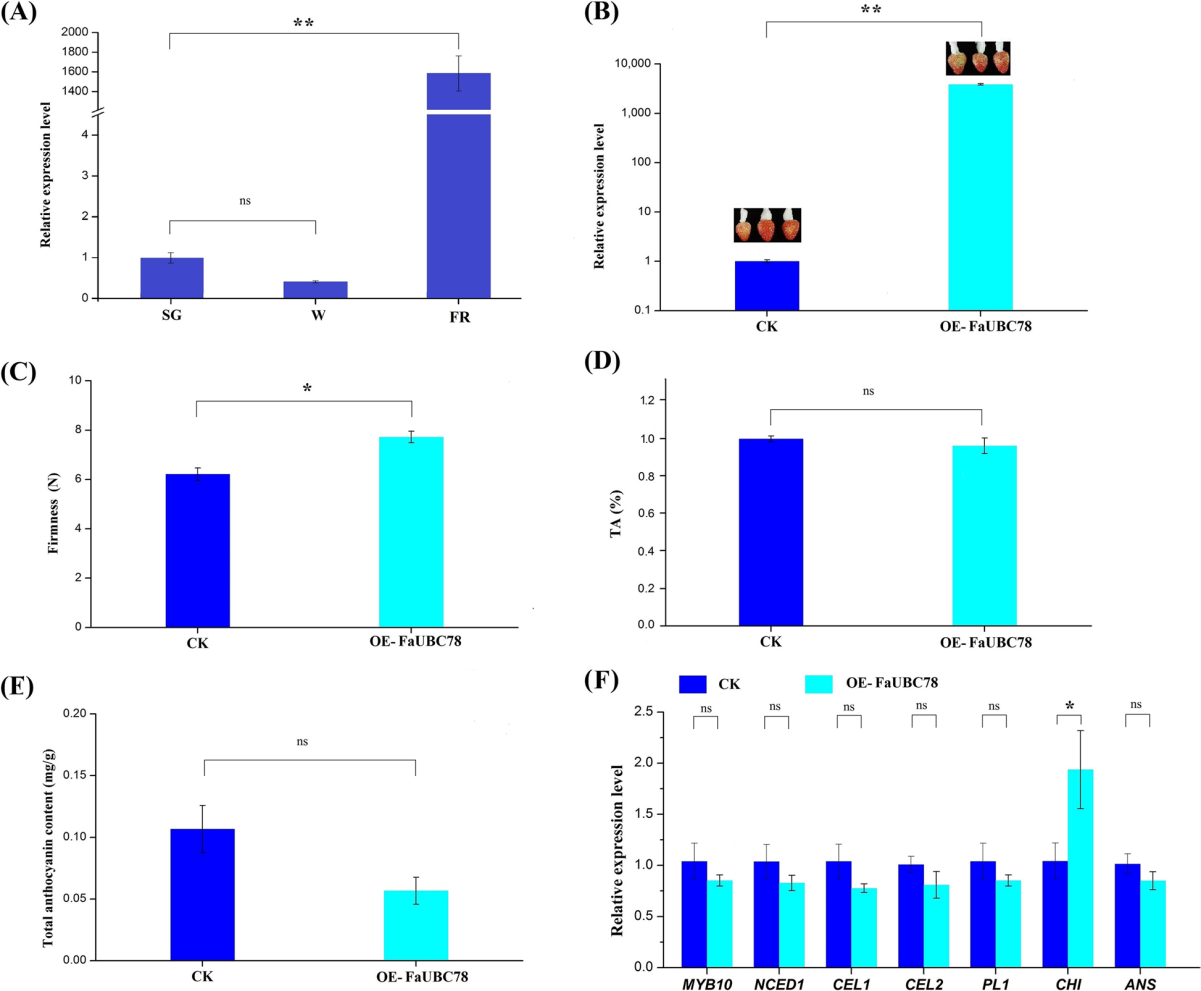
Overexpression for the *FaUBC78* gene in strawberry fruits. The injected fruits were harvested 8 days after injection. **A** RT-qPCR analysis of the relative expression level of *FaUBC78* at small green (SG), white (W) and partial red (PR) stages. **B** Transcript levels of *FaUBC78* in the control and overexpression fruit by RT-qPCR. **C**-**E** Fruit firmness, titratable acid content, anthocyanin content in the control and *FaUBC78* overexpression fruit. **F** Transcript levels of ripening-related genes in the control and *FaUBC78* overexpression fruits. Data are expressed as the mean ± SD of three replicates, and each repeat included 10 fruit individuals. Asterisks indicate significant differences (*P < 0.05, **P < 0.01, ns *P* > 0.05) between the means of the overexpression fruits and the control group

To validate the roles of FaUBC genes in strawberry fruit ripening, two FaUBC genes (*FaUBC76* and *FaUBC78*) included in the cluster 3 were selected for gene function analysis. A RT-qPCR analysis was performed to detect the UBC expression during fruit development stages. In agreement with the transcriptome data, the expression of *FaUBC76* and *FaUBC78* were abundantly expressed in FR, but at low levels in W stage (Figs. 9A and 10A). The two UBC genes were further validated by transient over-expression method.

At 5 days after injection, the fruit color of *FaUBC76* overexpression group was significantly redder than the control group (Fig. 9B). A significant higher expression level in the *FaUBC76* overexpression fruit than that of the control was observed, indicating the transient overexpression successfully resulted in the up-regulation of *FaUBC76*. The firmness of *FaUBC76* overexpression fruit was lower than that of the control group (Fig. 9C), whereas the TA were not significant (Fig. 9D). In addition, the content of total anthocyanins in overexpression fruit was higher than that of the control group (Fig. 9E). Furthermore, RT-qPCR was used to measure the expression of a set of ripening-related genes, including ABA biosynthesis gene (*NCED1*), cell wall-related genes (cellulase 1/2, *CEL1/2*, and pectin lyase 1, *PL1*), anthocyanin biosynthesis related genes (chalcone isomerase, *CHI*; anthocyanidin synthase, *ANS* and *MYB10*). The results showed that *FaMYB10, FaPL1, FaCEL1/2* were up-regulated, and *FaNCED1, FaANS* and *FaCHI* had no significant difference compared with that of the control fruit (Fig. 9E). These results indicated that *FaUBC76* is a positive regulatory factor that promotes strawberry ripening.

Between CK and *FaUBC78* overexpression fruit groups, the fruits were harvest after 8 days when the CK turned to fully red first. The relative expression level of *FaUBC78* was significantly increased in the overexpression fruit (Fig. 10B). Notably, the overexpression of *FaUBC78* significantly increased the firmness of strawberry fruit, but had no significant effects on fruit TA and anthocyanin content (Fig. 10C~E). RT-qPCR was used to measure the transcript levels of a set of ripening-related genes, and the result showed that only the expression level of *FaCHI* was significant up-regulated in the *FaUBC78* overexpressed fruit (Fig. 10F). These work reveals *FaUBC78* play a role in inhibiting the decrease of fruit firmness and delaying maturation time during fruit ripening.

## Discussion

In this study, a total of 191 UBC genes were identified in the genome of *Fragaria* ×*ananassa*. The number of FaUBC genes is far more than the 45, 48, 52, 75, and 74 UBC genes isolated from the genome of Arabidopsis [32], rice [5], tomato [6], banana [7] and maize [33], which indicates that the UBC family expanded in strawberry. Gene duplication events such as whole genome duplication, tandem duplication, segmental duplication, and transposition have a remarkable role in the expansion of gene family members in genomes [34], and increasing evidences has shown that whole genome duplication are largely responsible for the expansion of gene families in cultivated strawberry, such as the GST, GMP and RALF gene families [35–37]. Additionally, the genome sizes of Arabidopsis, strawberry, rice, tomato, maize, and banana are ~ 125, ~ 240, ~ 466, ~ 466, ~ 900, ~ 2300, and 523 Mb, respectively. Clearly, the higher number of UBC-encoding genes in strawberry can not only be explained by genome size.

Pear, peach, and strawberry are important fruit producing species belong to Rosaceae family, which share similarly genetic backgrounds. Previous studies also showed that there were more collinear regions in the genome, suggesting that the phylogenetic relationship of these three species was closely related [38]. In our study, a total of 69 and 74 UBC paralogous gene pairs were revealed in strawberry/peach and strawberry/pear, respectively, supporting a strong evolutionary relationship of UBC genes in Rosaceae species. The Ka/Ks value can further explain the gene selection pressure and evolution rate positive selection: Ka/Ks > 1 suggested positive selection, Ka/Ks = 1 suggested neutral selection, and Ka/Ks < 1 suggested negative selection [39]. All paralogous genes in UBC gene family among strawberry/peach and strawberry/pear have experienced purifying selection with the ratio of Ka/Ks less than one. These findings of collinearity analysis indicate that the genes were retained in Rosaceae species after whole genome duplication event and were strongly retained by stabilizing selection [40].

To further understand the regulatory network of UBC proteins, an interaction network was built. Most UBC proteins exhibited interactions with multiple proteins including several UBC proteins, RBX1, SUMO. A RING-H2 Protein, RBX1, bind the ubiquitin-conjugating enzyme E2 and bring it into close proximity with the E3 substrate [41]. And RCE1 (homolog of FaUBC3/4/9/15/22/28/31/37/39/43/45/49) which is interacts directly with RBX1 in Arabidopsis and play important role in early development [42]. There are other studies showing that an E2 ubiquitin ligase UBC-9 mediates covalent attachment of small ubiquitin-related modifier (SUMO) [43, 44]. This provided a useful reference for in-depth understanding the molecular interactions of FaUBC proteins.

Despite the potential functional significance of UBC members, only a few UBC family members have been described in horticultural plants with fruits as product organs, such as tomato [6], banana [7], papaya [45], grape [46] and pear [47]. Among them, several studies have been demonstrated that E2s are involved in the fruit ripening process. *SlUBC32*, which is upregulated during tomato (*Solanum lycopersicum*) fruit ripening and downregulated in the *rin* mutant, plays an important role in the regulation of fruit ripening [6]. Some of MaUBC genes are up- and downregulated during different ripening stages in banana (*Musa acuminata*) [7]. Thirteen (*CpUBC4*/*6*/*7*/*8*/*9*/*11*/*12*/*14*/*16*/*19*/*20*/*28*/*34*) and two (*CpUBC2* and *CpUBC10*) of the 34 *CpUBC* genes in papaya (*Carica papaya*) were up- or downregulated during the progression of fruit ripening, respectively [45]. To investigate the roles of *FaUBC* genes in the ripening of strawberry, the expression pattern of *FaUBC* genes were analyzed during the fruit ripening process. The results showed that cluster 3 exhibiting a sudden expression increase in the turning red stage were speculated to be involved in fruit ripening. Subsequently, two FaUBC genes (*FaUBC76* and *FaUBC78*) included in the cluster 3 were randomly selected for gene function analysis by transient over-expression method. The results showed that *FaUBC76* overexpression gave rise to higher anthocyanin content and lower firmness than the control. It has been documented that the color, firmness and soluble solids of fruit are important signs of strawberry ripening. For most fruit trees, fruit ripening is usually characterized by fruit softening and color transformation, accompanied by complex physiological and biochemical changes and volatile metabolism to form unique quality [48]. Furthermore, the gene expression analysis showed that *FaUBC76* overexpression can significantly increase gene expression of *FaMYB10*, *FaPL1, FaNCED1* and *FaCEL1/2* that involved in strawberry ripening. Therefore, we speculate that *FaUBC76* has a positive effect on the fruit development and ripening in strawberry. *FaUBC78* overexpression significantly enhance the strawberry firmness, but had no significant effects on fruit TA and anthocyanin content. Moreover, only the expression level of *FaCHI* was significant up-regulated in the *FaUBC78* overexpressed fruit, while other ripening-related genes had no significant change. Taken together, these findings suggested that the strawberry UBC family genes might be participated in the regulation of fruit development and ripening processes. However, only two genes of UBC family was studied in this experiment, so the regulation of other genes of the same family in strawberry fruit ripening needs to be further explored.

## Materials and methods

### Identification and comprehensive analysis of FaUBC genes

The UBC domain (PF00179) obtained from Pfam database was used as a query to search against the genome of strawberry downloaded from Genome Database for Rosaceae (GDR, https://www.rosaceae.org) [49]. The sequences with high score in the results were retrieved as putative UBC proteins. The number of amino acids, ORF length, putative protein molecular weights (MW) and isoelectric points (pI) for each sequence were obtained using a perl script. Multiple alignment was conducted using Geneious Prime software (https://www.geneious.com/). The conserved domains in the FaUBC proteins were screened and annotated based on Pfam [50], and NCBI-CDD [51]. The exon-intron structure of the FaUBC genes was analyzed using Gene Structure Display Server v.2.0 (http://gsds.cbi.pku.edu.cn/index.php). To identify the conserved motifs of FaUBC proteins, the MEME online program (http://meme-suite.org/tools/meme) was used with the following parameters: any number of repetition, maximum number of motifs was set as 10, and optimum motif length was set to 6-100 residues. The upstream 1500 bp regions of FaUBC genes were downloaded from the genome data and were performed to analyze the cis regulatory element using PlantCARE (http://bioinformatics.psb.ugent.be/webtools/plantcare/html/). The interaction networks of the identified proteins were analyzed by STRING (https://string-db.org/). GO and KEGG functional annotation of FaUBC proteins were performed by PANNZER2 (http://ekhidna2.biocenter.helsinki.fi/sanspanz/) and DAVID (https://david.ncifcrf.gov/), respectively.

### Phylogenetic and evolutionary analysis of UBC proteins

The Arabidopsis UBC protein sequences were downloaded from the Arabidopsis information source (TAIR) database, and the genomic data for pear (*Pyrus communis*) and peach (*Prunus persica*) were downloaded from Genome Database for Rosaceae (GDR, https://www.rosaceae.org). Phylogenetic tree of FaUBC proteins was constructed using Clustal X v.2.0 and MEGA v.7.0 software [52] with the neighbor-joining (NJ) method, and a bootstrap test with 1000 replicates were chose to evaluate the support of interior branches. The collinear gene pair was calculated using MCScanX software (http://chibba.pgml.uga.edu/mcscan2/), the synteny relationship was visualized by Circos (http://circos.ca/). Synonymous (Ks) and non-synonymous (Ka) substitutions per site between duplicated FaUBC gene pairs were subsequently calculated using KaKs Calculator v.1.2 software [53].

### Plant materials, RNA extraction, and cDNA synthesis

The strawberry (*Fragaria×ananassa* cv. Benihoppe) used as the experimental materials in our study were supplied by the Hanyuan County Dr. Luo Agriculture Co., Ltd. (Sichuan, China). The fruits at small green (SG), white (W) and full red (FR) stages were collected separately. These materials were quickly frozen and stored at -80 °C for subsequent experiments.

Total RNA was extracted from frozen strawberry fruit using the total RNA kit (Tiangen, Beijing, China) according to the manufacturer’s instructions. The RNA concentration was determined using a NanoDrop ND 2000 spectrophotometer, and purity was measured by the ratio OD260/OD280 (1.8-2.0) and OD260/OD230 (2.0-2.2). Finally, the integrity of the RNA was examined by 1.0% agarose gel electrophoresis. cDNA was synthesized from 1.0 μg of total RNA using the TransGene reverse transcription kits (Beijing, China). All cDNA samples were diluted 1:10 with RNase-free water for gene cloning and quantitative real-time PCR (RT-qPCR).

### Transcript abundance analysis and RT-qPCR analysis of FaUBC genes

The transcript abundance levels of FaUBC genes in strawberry were retrieved from the online transcriptomic data (SRA accession: SRX6381727). The expression cluster analysis was analyzed by TCseq R package.

RT-qPCR analysis was carried out using SYBR Green Premix Ex TaqTM (Takara, Japan) on a CFX96 qPCR system (Bio-Rad, USA) in triplicate of each sample. The relative expression was calculated using the 2^-ΔΔCt^ method [54]. *Fa26S* rRNA (accession: X58118) was used as the reference gene to standardize the raw data. All primers used in the present study were listed in Table S10.

### Transient overexpression of *FaUBC76* and *FaUBC78* in strawberry fruit

Full-length coding sequence of *FaUBC76* and *FaUBC78* was amplified using the cDNA sample. Then these two genes were cloned into the modified pCAMBIA1301 vector with CaMV35s promoter, respectively. The recombinant vectors was then introduced into the *Agrobacterium tumefaciens* strain GV3101. *Agrobacterium* infiltration was performed based on the previously described method [55]. The agrobacterium suspension was injected in the entire fruits at white (W) stage when they were still attached to the plants by a sterile 1 mL syringe. The injected fruits were harvested when they turned fully red after injection. As control, fruits at the same stage were injected with bacteria containing empty vector. Each treatment included three replicates, and each repeat included 10 fruit individuals.

### Determination of the TSS, firmness, the TA and total anthocyanin content

The fruit firmness was measured by using a digital display firmness tester (FR-5105, LUTRON, China). The total titratable acid (TA) content was measured by titration with 0.1 M NaOH titration, and expressed as a citric acid content percentage. Anthocyanin content determination was carried out by the pH-differential method using buffer solutions of sodium acetate (0.4 M, pH 1.0) and potassium chloride (0.025 M, pH 4.5), and the absorbance was read at 520 and 700 nm, respectively [56].

### Statistical analysis

The experiment was arranged in a completely randomized design with three replications. Statistical analysis was performed using multivariate logistic regression with the IBM SPSS statistics program (SPSS Version 27). Data were represented as average ± STDEV (*n* = 3). Analysis of variance (ANOVA) was used to determine the significant difference when *p* < 0.05.

## Supplementary Information

**Additional file 1: Figure S1.** Schematic gene structure and motif location of FaUBC genes.

**Additional file 2: Table S1.** The basic information of FaUBC genes in the genome of cultivated strawberry.

**Additional file 3: Table S2.** Collinear pairs identified between Arabidopsis and strawberry.

**Additional file 4: Table S3.** Ka, Ks and Ka/Ks of paralogous gene pairs of FaUBC genes.

**Additional file 5: Table S4.** Ka, Ks and Ka/Ks of paralogous gene pairs of UBC genes among three Rosaceae species.

**Additional file 6: Table S5.** Analysis of cis-regulatory elements in the promoter regions of FaUBC genes.

**Additional file 7: Table S6.** GO categories and distribution of FaUBC genes.

**Additional file 8: Table S7.** KEGG function classification of FaUBC genes.

**Additional file 9: Table S8.** The AtUBC proteins in protein-protein interaction network and the homologous FaUBC proteins in strawberry.

**Additional file 10: Table S9.** Expression of FaUBC genes and expression clusters.

**Additional file 11: Table S10.** Primers of ripening-related genes used to detect the expression level in overexpressed fruits.

## Data Availability

The following information was supplied regarding data availability: RNA-Seq data are available at the NCBI Sequence Read Archive: SRX6381727. The genome sequence data is available at GDR database (GDR, https://www.rosaceae.org).
