# Supplementary material for: Characterization and regulation mechanism analysis of ubiquitin-conjugating family genes in strawberry reveals a potential role in fruit ripening
Source: BMC Plant Biol. 2022 Jan 19;22:39. doi: 10.1186/s12870-021-03421-8 (PMC8767729; doi:10.1186/s12870-021-03421-8)
Supplement: Supplementary file 11 — Additional file 11: Table S10. Primers of ripening-related genes used to detect the expression level in overexpressed fruits. [file 12870_2021_3421_MOESM11_ESM.docx]

**Table S10. Primers of ripening-related genes used to detect the expression level in overexpressed fruits.**

| **Gene** | **Purpose** | **Amplicon size (bp)** | **Sequences of Forward & Reverse (5’-3’)** |  |
| --- | --- | --- | --- | --- |
| ***FaUBC76*** | transient overexpression | 447 | *F-:*G*CGGTGGCGGCCGCTCTAGACTCCTTCATCCTCAATTCTCATTCTCATTCTTCTTCAT*  *R-:ATCTGCAGCCCGGGGGATCCCCGAATCGATTACATGTTATGGAACTCTTCT* | |
| ***FaUBC78*** | transient overexpression | 978 | *F-:CGCGGTGGCGGCCGCTCTAGAATGGATGAAACTGCGTTCAAGC*  *R-:GATCTGCAGCCCGGGGGATCCCTAATTGAAGTTGGAATAAGGTCCTGG* | |
| ***FaUBC76*** | RT-qPCR | 218 | *F-GCGCTGCATGACGTAGGAG*  *R-* *CGATCTCTCTTCCTCTCTCTCTATCGC* | |
| ***FaUBC78*** | RT-qPCR | 244 | *F-CGGACACCATATACGTGCG*  *R-* *CTCCTCAATCCAAACCAGGTG* | |
| ***FaActin*** | RT-qPCR | 263 | *F*-TGGGTTTGCTGGAGATGAT  *R*-CAGTTAGGAGAACTGGGTGC | |
| ***FaMYB10*** | RT-qPCR | 173 | *F*-CAACAGCACCACCACAGACT  *R*-GCTTGCCGATTGTACCGTAT | |
| ***FaNCED1*** | RT-qPCR | 156 | *F*-CGGAACCTGCTCGGTAGAAA  R-AAGGAAGAAAGGCTCGCCAC | |
| ***FaCEL1*** | RT-qPCR | 83 | F-CCATACCCAAGTCCAATA  R-ACGATAGCGAAGTTACAT | |
| ***FaCEL2*** | RT-qPCR | 97 | F-TGATGCCTATGACAACTT  R-ACGAGCCAATATACCAATA | |
| ***FaPL1*** | RT-qPCR | 166 | F-TGACTCCCTTGCTTCTT  R-TCTACTGCGTGCTCATTCCA | |
| ***FaCHI*** | RT-qPCR | 89 | F-AGCGAAAGCCATTGAAAAGT  R-CATTTGGTGATTGTGTGAAGAG | |
| ***FaANS*** | RT-qPCR | 195 | F-CGTGAGACCCAAAGAGGA R-ATGCCGTGGTTGATAAGG | |
